# The Role of Academic Buoyancy and Social Support on English as a Foreign Language Learners’ Motivation in Higher Education

**DOI:** 10.3389/fpsyg.2022.892603

**Published:** 2022-05-20

**Authors:** Yanxian Jia, Ling Cheng

**Affiliations:** ^1^School of Literature, Media, and Education Studies, Chaohu University, Hefei, China; ^2^School of Humanities and International Education and Exchange, Anhui University of Chinese Medicine, Hefei, China

**Keywords:** academic buoyancy, English as a foreign language, motivation, social support, psycho-education

## Abstract

Since motivation determines progress in the class, it has a significant role in the field of learning English as a foreign language (EFL), contributing to a successful learning process. Therefore, teachers need to motivate learners to achieve their learning goals and provide them with a meaningful learning process in stressful situations. Two factors are required to successfully overcome challenges in scholastic circumstances, such as academic buoyancy and social support. The former can be a significant element in a psycho-educational setting that helps learners with academic difficulties and the latter is another term that is deemed as an important predictor of academic motivation even when considering perceived support from teachers and peers. The functions of academic buoyancy and social support factors in the process of learning and their association with students’ motivation have not been taken into account so far. As a result, this review has implications for scholars, theorists, and practitioners in quest of better investigating and addressing the roles of buoyancy and social support on students’ motivation.

## Introduction

In line with recent investigations, motivation is an important element in beginning and sustaining a learners’ educational cycle in the language teaching context (Randhawa and Gupta, 2000; Wigfield et al., 2015) and it has been utilized to describe how much interest and endeavor learners spend on educational exercise. In activities related to teaching and learning, motivation is regarded as a significant part of making learners focus on the material learned in the classroom (Dahliana, 2019) that is characterized as an integral cycle in the presentation and prolongation of exercises devoted to a particular objective (Wigfield et al., 2015). The notion of motivation is utilized to clarify how much interest and endeavor learners spend on educational exercises in the language education context. Furthermore, there is no lack of proof that motivation is the foundation of effective language education (Dörnyei and Ryan, 2015). Also, there is no dearth of proof that motivation supports the effective fulfillment in language education (Dörnyei and Ryan, 2015). Nonetheless, studies on the social-psychological dimensions of education have demonstrated that, when conceptualized in ways that do not consider the actuality of constant difficulties, failures, and tension, motivation on its own is not enough to justify effective educational manners and scholastic presentation (Kaplan et al., 2012).

In numerous contexts, the daily actuality of guided language education can lead to fluctuations, tensions, and even failures (Bailey and Phillips, 2016; Bücker et al., 2018). In these situations, the daily reality of instructed language learning contains ups and downs, pressures, and even obstacles, such as poor grades, test anxiety and stress, and failures in presentation, deadlines, scholastic competitions, tensions associated with tests, and school issues (Martin and Marsh, 2008). Therefore, since most learners have a difficult time while trying for achievement, it can be said that motivation is essential to their achievement, and the benefits that learners obtain could be diminished if they do not maintain it, stay unaffected by failures and difficulties, and conquer the tensions that are a regular component of language education (Ushioda, 2008).

Moreover, the concept of academic buoyancy has been arisen with the growth of positive psychology to help learners cope with the daily misfortunes encountered in their scholastic life (Martin and Marsh, 2009; Yu et al., 2019; Wang et al., 2021) that has been designated as a significant trait helping learners to cope with academic risks (Putwain et al., 2012), and it has been determined as an element that practitioners can nurture to assist learners with positively directing scholastic challenges as they emerge (Collie et al., 2017a). According to some studies, learners who can successfully deal with common failures at school, like difficult tasks or setbacks in tests, work in the best manner and accomplish more (Martin, 2014; Collie et al., 2015). In the same vein, academic buoyancy involves developing personal strong points by focusing on an issue as opposed to a responsive method during failures and difficulties in the educational domain (Martin, 2014). Since language education possesses all the attributes of a topic that necessitates buoyancy for eventual educational achievement, buoyancy goes along with motivation and is to a certain extent classified in that category; buoyancy maintains motivation, and thus, gives students the ability to cope with the fluctuations of daily language education, to continue their endeavors and conquer failures on the road to language educational achievement (Martin et al., 2010).

One of the most important elements in peoples’ survival when dealing with difficulties is social support (Ronen et al., 2016; Agbaria et al., 2017). People regularly need help from others to conquer difficulties in learning and social life and to live a sustained successful life and in the literature, these support factors are alluded to as social support factors (Atnafu, 2012). Besides individual buoyancy, social support is a vital factor concerning learners’ scholastic presentation and school involvement (Martin et al., 2017), and commitment (Skinner et al., 2008). Individuals attain various types of information through social support as they attain information that makes them have faith in the fact that others appreciate and are fond of them as well as information that makes them have faith that they are valued and a part of a social network and reciprocal involvement (Agbaria and Bdier, 2020). Educators have an important function in this context as they not only convey their information to their learners but also carry out their obligations. Educators require specific abilities in key assignments like teaching, instructing, directing, coaching, evaluating, and appraising learners in the formal learning context (Pishghadam et al., 2021). Wentzel (2016) referred to multi-functional or multi-role terminologies concerning the issue and educators provide learners with tasks that necessitate their diligent participation in the content, instill high beliefs in them, frequently administer top priorities for scholastic objectives, and develop a tremendous school setting or culture.

Social support offers influential assets (offering guidance), informative (giving direction), and/or emotional (taking care of a person). These assets assist individuals with dealing with difficulties in their lives and with being more sympathetic and reactive to others and wellbeing and joy are increased through emotional, influential, and informative support (Morelli et al., 2015). The main aspect of backing a learner’s scholastic success is the existence of helpful human connections. About scholastic anxiety, social support alludes to associations within social networks that assist people with discovering answers to scholastic issues at school. Effective learners are prone to have a helpful network, encounter little scholastic misfortune, and may stay academically buoyant during failures and difficulties at school (Martin, 2014; Collie et al., 2017b). Social support involves emotional, psychological, and physical safety that comes from relatives, companions, and significant individuals (Mega et al., 2014). Studies show that social support from educators, colleagues, and guardians can help improve scholastic performance in adolescence and stop mental deterioration (Malecki and Demaray, 2006). Elements from these origins with which people associate are major facilitators of the combination of outer and inner motivations (Muller and Palekcic, 2005).

Regarding the function of motivation in language education, a group of scholars (Change, 2010; Wong et al., 2013) has noted the connection between social support and students’ motivation. Also, it was discovered that the educators’ support affected the subjects’ motivation to study English that means social support plays a significant role in the constructive growth of youngsters as well as in scholastic motivation (Oberle et al., 2011; Collie et al., 2016). Among these social support sources, according to research, educators who offer learners maximum levels of independent support are more prone to describing the importance of educational exercises, developing learner-centric settings, motivating learners’ creativity, going after learners’ wants and requirements, and trying to comprehend learners’ emotive conditions, than those who offer minimum levels of independent support (Assor et al., 2002). Moreover, past investigations have proved that learners’ academic buoyancy is connected to maximum results like scholastic success, involvement, and health (Miller et al., 2013; Martin, 2014; Martin et al., 2017; Datu and Yang, 2018).

According to literature reviews, learners who mentioned that they constantly experienced troublesome circumstances in their class (that is circumstances in which they encounter misunderstandings, setbacks, and great degrees of hardship) have lower levels of school pleasure than learners who do not constantly experience such circumstances (Vedder et al., 2005). Their self-esteem, motivation, and school adaptation are deconstructivity connected to the prevalence of troublesome circumstances. However, based on the researcher’s knowledge, little is conveyed about the role of buoyancy and social support in the foreign education domain on the one hand and, its role in enhancing motivation, on the other hand. Consequently, one of the notable purposes of the current review was to take the issue into account in language learning and research and examine their central functions on language learners’ motivation.

## Review of the Literature

### Academic Buoyancy

Academic buoyancy is a notion close to emotive management. Buoyancy alludes to an assessment of a person’s ability to cope with failures. Emotions, however, allude to mental cycles that occur as a reaction to anxious or worrisome occurrences (Putwain et al., 2012; Collie et al., 2015). Buoyancy in academic environments is essential in affecting academic engagement and minimizing the impact of academic anxiety on learner engagement (Martin and Marsh, 2009). Buoyancy is associated with daily problems and worries that disrupt students’ motivation and involvement in the process of learning by threatening their confidence and persistence (Martin et al., 2017). As a related concept, resilience qualitatively varies from buoyancy since it pertains more closely to intense adversity or debilitating threats to the growth, that is, persistent separation and self-disability, opposition or alienation from others in the school setting (Martin and Marsh, 2009).

In comparison with the construct about academic resilience, buoyancy concentrates on mitigated and temporary difficulties in school life, like experiencing isolated weak performance (Martin and Marsh, 2009). When it comes to the dimension of interference that notices the contrasts between flexibility and buoyancy, one may think that academic buoyancy is a needed, yet inadequate prerequisite for scholastic flexibility. This means that flexible learners are prone to be buoyant, insinuating a chain of command. Therefore, it is important to help learners cope with constant difficulties and needs to build their buoyancy, to promote learners’ flexibility to more notable unfavorable scholastic and life occurrences. Buoyancy is the initial requirement for increasing flexibility and assisting people with counterbalancing risk (Martin and Marsh, 2009). There is proof insinuating that academic buoyancy influences not only academic and mental result measures where the former alludes to the pleasure of attending school and the classroom and the latter including overall self-confidence and self-effectiveness (Martin et al., 2010).

### Social Support

Both intrinsic and extrinsic motivations are determined by social factors (Ryan and Deci, 2020). Factors, with which an individual interacts, such as family, teachers, friends, and classmates, are important factors in integrating extrinsic and intrinsic motivations (Muller and Palekcic, 2005). Social support provided to individuals is related to extrinsic motivation but is also likely to be internalized (Ryan and Deci, 2020). The construct social support alludes to the social and mental support one attains or discerns in their setting, including trusting others for direction and help and revelation of issues (Taylor, 2011; Chang et al., 2020). Social support is characterized as the offering of physical, emotive, knowledge, and instrumental help that a person discerns from a social network and it includes a variety of societal associations with partners, extended relatives, companions, and so on (Siklos and Kerns, 2006). The concept of social support is usually categorized into constitutional and practical support. While the latter is related to the discerned standard of social connections, the former is related to the presence and number of connections (Hefner and Eisenberg, 2009). Social support can fall under four classes, from a relational viewpoint. Emotional support alludes to showing empathy, kindness, fondness, and faith in others. Instrumental support alludes to offering content support and services when others require them. Assisting others with overcoming issues by offering beneficial proposals, information, and so on, is known as knowledge support. Offering beneficial information that helps others assess themselves is referred to as evaluation support (Taylor, 2011).

Social support is a comprehensive concept that includes various notions that can be categorized into two kinds. Firstly, impartial and perceivable support, namely, content support and network support (well-balanced societal connections like matrimony, peers, companions, etc., and unbalanced social connections, like casual circles and so on) that do not rely on personal conceptions and are therefore an impartial actuality. Secondly, personal support, that is, emotive support and a sense of regard and compassion by a person in societal life, is similar to a person’s emotions (Haber et al., 2007). Emotional support from teachers and academic support are critical to learners’ success (Patrick et al., 2007) and it includes the perception of the instructor’s care and love for the learners, while academic support is in charge of the learners’ learning strategies and study skills. Studies show that to be effective in the classroom, instructor support must be fully aligned with learners’ efforts, classroom discipline, and the application of self-determination strategies (Dearnley and Matthew, 2007; Wentzel et al., 2010). Learners without optimal social support are at higher risk for less adaptive learning outcomes when they struggle with academic difficulties (Collie et al., 2017b). Based on social support theories, educational research hypothesizes that supportive relationships enhance learners’ sense of competence, which can lead to higher motivation in school (Skinner et al., 2008). Social support can efficiently aid learners handle challenging situations and lower their mental stress. It supports the development of mental health by preventing mental health problems (Chang et al., 2020). Relatives and teachers as other sources of social support can also have an overall positive impact on learners’ willingness to take risks and improve their physical and mental health (Zhou, 2020).

### Motivation

According to Gopalan et al. (2018) for a long time, building motivation has been seen as the key to success and progress for people in their personal and academic lives and it is a driving factor behind any tasks people do without which everything becomes possible. Different perspectives of education have extensively studied motivation from various aspects and perspectives, all confirming the effect of such construct on the students’ success and learning (Dörnyei and Ushioda, 2013; Al-Hoorie, 2017). The construct “motivation” is frequently utilized to explain something that helps individuals move, keep moving, and get things done (Pintrich, 2003). Academic motivation, which refers to the learners’ willingness to learn efficiently in a learning environment, is associated with learners’ academic outcomes (Green et al., 2012). In addition, a force that can manage, encourage, and promote goal-directed behavior is the motivation that is not only important for the learning process but also academic success (Kosgeroglu et al., 2009).

Theory of extrinsic and intrinsic motivation regards motivation pushed by internal and external powers. When people engage in an activity solely for pleasure and entertainment, their motivation is intrinsically formed. In addition, when an individual’s motivation to finish a task is to obtain an extrinsic prize or admire, it is held that he has extrinsic motivation (Brown and Lee, 2015). This theory holds that there are positive and negative aspects to both types of motivation. However, intrinsic motivation has higher effectiveness during the challenging language learning process because it inspires students to interact, work hard, and achieve long-term academic achievement (Pinder, 2011). A person’s involvement in an exercise based on the person’s pleasure and attention during the exercise regardless of anticipating an outer prize is inner motivation (Ryan and Deci, 2000). It is the act of a person due to an inner ordinary appeal without an outer impetus. Outer motivation, however, is related to a person’s involvement in an exercise to attain a prize (Ryan and Deci, 2000; Atnafu, 2012). Motivation in the classroom is affected by at least five factors: students, teachers, class content, method of teaching, and learning setting (D’Souza and Maheshwari, 2010). Furthermore, motivation helps to academic success, even when cognitive abilities are jointly taken into account (Wigfield and Wentzel, 2007).

## Conclusion

As colleges go through important enhancements and alterations, learners’ motivation should be addressed. Motivation is a primary non-intellectual mental element that causes the enhancement of learners’ scholastic presentation and it is also a primary element in assisting with learners’ preparation for life after school. In this way, the present review may stimulate numerous implications concerning both hypothetical and educational viewpoints. Based on the literature review, future achievements and great degrees of scholastic motivation will take place by inspiring buoyant learners and increasing social support. This can be manifested by educators, learners, material developers, scholastic professionals, and college administrators in higher education. Colleges are a place where scholastic difficulties, failures, and tensions are widespread in people’s lives and despite the existence of daily scholastic misfortunes, there are quite a few learners who successfully face and solve these difficulties (Martin et al., 2010). Shielded by a collaborative and supportive setting and increased feeling of dominance over scholastic assignments, adversities can be sometimes lead to an inclination in learners to increase their efforts and motivation; thus, academic buoyancy and social support are significant in this regard (Martin and Marsh, 2008). Learners who are buoyant and greatly supported by family and community are more motivated to adapt (Collie et al., 2017b) and such a high level of motivation also explains the learners’ academic success (Kriegbaum et al., 2018).

The significance of social context in forming learner motivation is guaranteed in theoretical perspectives and motivation theories like self-determination and the theory of social cognitive (Camacho et al., 2021). For instance, the school environment affects learners’ motivation directly or indirectly, and as a result brings about their involvement (Anderman and Gray, 2017). It was found that perceived social support in teachers remains an important indicator of academic motivation even when the perceived support of parents and peers is taken into account (Wentzel, 2009). Social support is taken into account as something which enhances the motivation required to gain success and is effectively influential in coping with stressful situations (Tezci et al., 2015). Educators ought to keep in mind their role as the main social support source for learners who can create a structured, cooperative, and autonomous supportive classroom setting that meets learners’ underlying psychological requirements and academic motivation (Ryan and Deci, 2020). According to Demir (2019), learners are more likely to attend class when they are supported by their teachers, and regular attendance with more time to build close relationships with teachers can contribute to consistent attendance and improvement of motivation. Furthermore, social support has an important function in reducing distress because it improves self-value, societal self-esteem, and the sense that they can manage the flow of life (Besser and Priel, 2008). Social support alludes to social resources, assets, or networks that individuals can utilize when they require help, guidance, backing, reinforcement, acceptance, consolation, security, or succor. It outlines the knowledge that one is liked, respected, cherished, and part of a network of connections and reciprocated responsibility (Vedder et al., 2005). Social support can be appreciated by learners in the setting of education and teaching, which results in motivation, collaboration, and adaptation to school.

## Implications and Future Directions

In academic motivation, the positive role of social support is proved, indicating a positive relationship between learners’ academic motivation and support from parents, instructors, and friends (Atnafu, 2012; Tezci et al., 2015). The syllabus designers should design tasks in a way that can help the combination of partnership and social association, in cooperation with attentive attention to educational difficulties that consequently bring about opportunities to foster motivation. The possible clarification for this result is that social support enhances constructive feelings and joy, assists people with dealing with the causes of anxiety, offers necessary knowledge and guidance, and gives people their value, thereby elevating a sense of self-confidence and self-competence (Agbaria, 2013). Instructors can take advantage of the opinions offered in this review article as they learn about the significance of support and its effect on different dimensions of student education. Through social support, proper interactive rapport can encourage learners to consider the instructor not only as a teacher but also as a friend able to aid learn the course content. In this way, teacher–student interactions can be successful, and instructors can fulfill their roles in the perfect term.

The review of literature proposed that an element that practitioners can develop to assist learners with positively overcoming scholastic challenges when they emerge is academic buoyancy (Collie et al., 2017a) and it allows students to stay motivated despite failures and hard encounters and transform this willpower into positive educational results in a teaching environment. The function of academic buoyancy is to describe the competence of students in staying constructively motivated, remaining unaffected by failures and difficulties, and solving the tensions that are common in learning. Buoyancy development exercises can become available to all learners when it comes to social support. The role of a positive and supportive classroom atmosphere can be promoted when teaching mindfulness and encouraging to identify educational stress and aptitudes to handle it at the individual level. Research literature (Miller et al., 2013) indicates that learners’ academic buoyancy was associated with significant motivational results, namely, higher persistence, positive emotional results, academic success, wellbeing, and academic performance. A deeper comprehension of academic buoyancy can help educational policymakers, educators, and scholars promote positive results (Miller et al., 2013). Furthermore, teachers should provide opportunities for the students to be confident enough in the classroom even in case of challenges. Correspondingly, teachers are supposed to support their learners in the process of learning and provide them with techniques that can encourage them to be more engaged in the classroom that result in more motivation and achievement. Likewise, the faculty educators can offer courses and workshops that address the theoretical and practical aspects of scholastic support for teachers to advance their potential in this realm. Additionally, more research with diverse research designs can be conducted to contain data associated with the variables of the present research, such as motivation to provide a comprehensive insight into the issue. Employing some kinds of interventions is suggested in further studies in clarifying the issue in a language context.

## Author Contributions

YJ conceptualized and drafted the initial manuscript. LC proofread and approved the final version. both authors have made a substantial, direct,and intellectual contribution to the work.

## Funding

This study is sponsored by the University-level scientific research projects of Chaohu University “Investigation and improving approaches of College students wellbeing under the pandemic in Anhui Province” (Grant No. XWY-202134) and the Key University-level pedagogical projects funded by Chaohu University (Grant No. CH21JXYJ08).

## Conflict of Interest

The authors declare that the research was conducted in the absence of any commercial or financial relationships that could be construed as a potential conflict of interest.

## Publisher’s Note

All claims expressed in this article are solely those of the authors and do not necessarily represent those of their affiliated organizations, or those of the publisher, the editors and the reviewers. Any product that may be evaluated in this article, or claim that may be made by its manufacturer, is not guaranteed or endorsed by the publisher.

## References

[ref1] Agbaria Q. (2013). Depression among Arab students in Israel: The contribution of religiosity, happiness, social support and self control. Soc. Stud. 3, 721–738.

[ref2] Agbaria Q. Bdier D. (2020). The role of self-control, social support and (positive and negative afects) in reducing test anxiety among Arab teenagers in Israel. Child Indic. Res. 13, 1023–1041. doi: 10.1007/s12187-019-09669-9

[ref3] Agbaria Q. Mahamid F. Ziya Berte D. (2017). Social support, self-control, religiousness and engagement in high risk-behaviors among adolescents. Int. J. Ind. Psychol. 4, 13–33. doi: 10.25215/0404.142

[ref4] Al-Hoorie A. H. (2017). Sixty years of language motivation research: looking back and looking forward. SAGE Open 7, 1–11. doi: 10.1177/2158244017701976

[ref5] Anderman E. M. Gray D. L. (2017). “The roles of schools and teachers in fostering competence motivation,” in Handbook of Competence and Motivation: Theory and Application. eds. Elliot A. J. Dweck C. S. Yeager D. S. (New York, NY: The Guilford Press), 604–619.

[ref6] Assor A. Kaplan H. Roth G. (2002). Choice is good, but relevance is excellent: autonomy-enhancing and suppressing teacher behavior’s predicting students’ engagement in schoolwork. Br. J. Educ. Psychol. 72, 261–278. doi: 10.1348/000709902158883, PMID: 12028612

[ref7] Atnafu M. (2012). Motivation, social support, alienation from the school and their impact on students’ achievement in mathematics: The case of tenth grade students. Ethiopian J. Educ. Sci. 8, 53–74.

[ref8] Bailey T. H. Phillips L. J. (2016). The influence of motivation and adaptation on students’ subjective well-being, meaning in life and academic performance. High. Educ. Res. Dev. 35, 201–216. doi: 10.1080/07294360.2015.1087474

[ref9] Besser A. Priel B. (2008). Attachment, depression, and fear of death in older adults: The roles of neediness and perceived availability of social support. Personal. Individ. Differ. 44, 1711–1725. doi: 10.1016/j.paid.2008.01.016

[ref10] Brown H. D. Lee H. (2015). Teaching by Principles: An Interactive Approach to Language Pedagogy. 4th Edn. White Plains, NY: Pearson Education.

[ref11] Bücker S. Nuraydin S. Simonsmeier B. A. Schneider M. Luhmann M. (2018). Subjective well-being and academic achievement: A meta-analysis. J. Res. Pers. 74, 83–94. doi: 10.1016/j.jrp.2018.02.007

[ref12] Camacho A. Correia N. Zaccoletti S. Daniel J. R. (2021). Anxiety and social support as predictors of student academic motivation during the COVID-19. Front. Psychol. 12:644338. doi: 10.3389/fpsyg.2021.644338 34108910 PMC8183683

[ref13] Chang Q. Peng C. Guo Y. Cai Z. Yip P. S. (2020). Mechanisms connecting objective and subjective poverty to mental health: serial mediation roles of negative life events and social support. Soc. Sci. Med. 265:113308. doi: 10.1016/j.socscimed.2020.113308, PMID: 32905965

[ref14] Change G. E. (2010). Redd-plus, forest people’s rights and nested climate governance. Glob. Environ. Chang. 20, 423–425. doi: 10.1016/j.gloenvcha.2010.04.007

[ref15] Collie R. J. Ginns P. Martin A. J. Papworth B. (2017a). Academic buoyancy mediates academic anxiety’s effects on learning strategies: An investigation of English- and Chinese-speaking Australian students. Educ. Psychol. 37, 947–964. doi: 10.1080/01443410.2017.1291910

[ref16] Collie R. J. Martin A. J. Bottrell D. Armstrong D. Ungar M. Liebenberg L. (2017b). Social support, academic adversity and academic buoyancy: A person-centred analysis and implications for academic outcomes. Educ. Psychol. 37, 550–564. doi: 10.1080/01443410.2015.1127330

[ref17] Collie R. J. Martin A. J. Malmberg L. E. Hall J. Ginns P. (2015). Academic buoyancy, student’s achievement, and the linking role of control: A cross-lagged analysis of high school students. Br. J. Educ. Psychol. 85, 113–130. doi: 10.1111/bjep.12066, PMID: 25604513

[ref18] Collie R. J. Martin A. J. Papworth B. Ginns P. (2016). Students’ interpersonal relationships, personal best goals, and academic engagement. Learn. Individ. Differ. 45, 65–76. doi: 10.1016/j.lindif.2015.12.002

[ref19] D’Souza K. A. Maheshwari S. K. (2010). Factors influencing student performance in the introductory management science course. Acad. Educ. Leadership J. 14, 99–119.

[ref20] Dahliana S. (2019). Students’motivation and responsive pedagogy in language classroom. Eng. J. Lang. Educ. Hum. 6, 75–87. doi: 10.22373/ej.v6i2.4601

[ref21] Datu J. A. D. Yang W. (2018). Psychometric validity and gender invariance of the academic buoyancy scale in the Philippines: A construct validation approach. J. Psychoeduc. Assess. 36, 278–283. doi: 10.1177/0734282916674423

[ref22] Dearnley C. Matthew B. (2007). Factors that contribute to undergraduate student success. Teach. High. Educ. 12, 377–391. doi: 10.1080/13562510701278740

[ref23] Demir S. (2019). A structural model on the role of perceived multi-dimensional social support in attitudinal variables. European. J. Educ. Res. 8, 607–616. doi: 10.12973/eu-jer.8.2.607

[ref24] Dörnyei Z. Ryan S. (2015). The Psychology of the Second Language Learner Revisited. New York, NY: Routledge.

[ref25] Dörnyei Z. Ushioda E. (2013). Teaching and Researching: Motivation. 2nd Edn. London: Routledge.

[ref26] Gopalan S. Cherikh M. Balakrishnan L. (2018). An exploratory investigation of motivation orientations of Indian business students: implications for educators. Int. J. Ind. Bus. Manage. 17, 455–477. doi: 10.1504/IJICBM.2018.095679

[ref27] Green K. W. Zelbst P. J. Meacham J. Bhadauria V. S. (2012). Green supply chain management practices: impact on performance. Supply Chain Manage. Int. J. 17, 290–305. doi: 10.1108/13598541211227126

[ref28] Haber M. G. Cohen J. L. Lucas T. Baltes B. B. (2007). The relationship between self-reported received and perceived social support: a meta-analytic review. Am. J. Community Psychol. 39, 133–144. doi: 10.1007/s10464-007-9100-9, PMID: 17308966

[ref29] Hefner J. Eisenberg D. (2009). Social support and mental health among college students. Am. J. Orthopsychiatry 79, 491–499. doi: 10.1037/a0016918 20099940

[ref30] Kaplan A. Katz I. Flum H. (2012). “Motivation theory in educational practice: knowledge claims, challenges, and future directions,” in Apa Educational Psychology Handbook: Individual Differences and Cultural and Contextual Factors. eds. Harris K. Graham S. Urdan T. Graham S. Royer J. Zeidner M. (Washington, DC: American Psychological Association), 165–194.

[ref31] Kosgeroglu N. Acat M. B. Ayranci U. Ozabaci N. Erkal S. (2009). An investigation on nursing, midwifery and health care students’ learning motivation in Turkey. Nurse Educ. Pract. 9, 331–339. doi: 10.1016/j.nepr.2008.07.003, PMID: 18768371

[ref32] Kriegbaum K. Becker N. Spinath B. (2018). The relative importance of intelligence and motivation as predictors of school achievement: A meta-analysis. Educ. Res. Rev. 25, 120–148. doi: 10.1016/j.edurev.2018.10.001

[ref33] Malecki C. K. Demaray M. K. (2006). Social support as a buffer in the relationship between socio-economic status and academic performance. Sch. Psychol. Q. 21, 375–395. doi: 10.1037/h0084129

[ref34] Martin A. J. (2014). Academic buoyancy and academic outcomes: towards a further understanding of students with attention-deficit/hyperactivity disorder, students without ADHD, and academic buoyancy itself. Br. J. Educ. Psychol. 84, 86–107. doi: 10.1111/bjep.12007, PMID: 24547755

[ref35] Martin A. J. Colmar S. H. Davey L. A. Marsh H. W. (2010). Longitudinal modelling of academic buoyancy and motivation: do the 5Cs hold up over time? Br. J. Educ. Psychol. 80, 473–496. doi: 10.1348/000709910X486376, PMID: 20170601

[ref36] Martin A. J. Marsh H. W. (2008). Academic buoyancy: towards an understanding of students’ everyday academic resilience. J. Sch. Psychol. 46, 53–83. doi: 10.1016/j.jsp.2007.01.002, PMID: 19083351

[ref37] Martin A. J. Marsh H. W. (2009). Academic resilience and academic buoyancy: multidimensional and hierarchical conceptual framing of causes, correlates, and cognate constructs. Oxf. Rev. Educ. 35, 353–370. doi: 10.1080/03054980902934639

[ref38] Martin A. J. Yu K. Ginns P. Papworth B. (2017). Young people’s academic buoyancy and adaptability: A cross-cultural comparison of China with North America and the United Kingdom. Educ. Psychol. 37, 930–946. doi: 10.1080/01443410.2016.1202904

[ref39] Mega C. Ronconi L. De Beni R. (2014). What makes a good student? How emotions, self-regulated learning, and motivation contribute to academic achievement. J. Educ. Psychol. 106, 121–131. doi: 10.1037/a0033546

[ref40] Miller S. Connolly P. Maguire L. K. (2013). Wellbeing, academic buoyancy and educational achievement in primary school students. Int. J. Educ. Res. 62, 239–248. doi: 10.1016/j.ijer.2013.05.004

[ref41] Morelli S. A. Lee I. A. Arnn M. E. Zaki J. (2015). Emotional and instrumental support provision interact to predict well- being. Emotion 15, 484–493. doi: 10.1037/emo0000084, PMID: 26098734 PMC4516598

[ref42] Muller F. H. Palekcic M. (2005). Continuity of motivation in higher education: A three-year follow-up study. Rev. Psychol. 12, 31–43.

[ref43] Oberle E. Schonert-Reichl K. Zumbo B. (2011). Life satisfaction in early adolescence: personal, neighborhood, school, family, and peer influences. J. Youth Adolesc. 40, 889–901. doi: 10.1007/s10964-010-9599-1, PMID: 21042841

[ref44] Patrick H. Ryan A. M. Kaplan A. (2007). Early adolescents’ perceptions of the classroom social environment, motivational beliefs, and engagement. J. Educ. Psychol. 99, 83–98. doi: 10.1037/0022-0663.99.1.83

[ref45] Pinder W. C. C. (2011). Work Motivation in Organizational Behavior, New York, NY: Taylor and Francis.

[ref46] Pintrich P. R. (2003). “Motivation and classroom learning,” in Handbook of Psychology: Educational Psychology. eds. Reynolds W. M. Miller G. E. (Hoboken, NJ: Wiley), 103–122.

[ref47] Pishghadam R. Derakhshan A. Jajarmi H. Tabatabaee Farani S. Shayesteh S. (2021). Examining the role of teachers’ stroking behaviors in EFL learners’ active/passive motivation and teacher success. Front. Psychol. 12:707314. doi: 10.3389/fpsyg.2021.707314, PMID: 34354648 PMC8329251

[ref48] Putwain D. W. Connors L. Symes W. Douglas-Osborn E. (2012). Is academic buoyancy anything more than adaptive coping? Anxiety Stress Coping Int. J. 25, 349–358. doi: 10.1080/10615806.2011.582459, PMID: 21644112

[ref49] Randhawa B. S. Gupta A. (2000). Cross-national gender differences in mathematics achievement, attitude, and self-efficacy within a common intrinsic structure. Can. J. Sch. Psychol. 15, 51–66. doi: 10.1177/082957350001500205

[ref50] Ronen T. Hamama L. Rosenbaum M. Mishely-Yarlap A. (2016). Subjective well-being in adolescence: The role of self-control, social support, age, gender, and familial crisis. J. Happiness Stud. 17, 81–104. doi: 10.1007/s10902-014-9585-5

[ref51] Ryan R. M. Deci E. L. (2000). Self-determination theory and the facilitation of intrinsic motivation, social development, and well-being. Am. Psychol. 55, 68–78. doi: 10.1037/110003-066X.55.1.68, PMID: 11392867

[ref52] Ryan R. M. Deci E. L. (2020). Intrinsic and extrinsic motivation from a self-determination theory perspective: definitions, theory, practices, and future directions. Contemp. Educ. Psychol. 61:101860. doi: 10.1016/j.cedpsych.2020

[ref53] Siklos S. Kerns K. A. (2006). Assessing need for social support in parents of children with autism and Down syndrome. J. Autism Dev. Disord. 36, 921–933. doi: 10.1007/s10803-006-0129-7, PMID: 16897397

[ref54] Skinner E. A. Furrer C. Marchand G. Kindermann T. (2008). Engagement and disaffection in the classroom: part of a larger motivational dynamic? J. Educ. Psychol. 100, 765–781. doi: 10.1037/a0012840

[ref55] Taylor S. E. (2011). “Social support: A review,” in The Oxford Handbook of Health Psychology. ed. Friedman H. S. (New York, NY: Oxford University Press), 189–214.

[ref56] Tezci E. Sezer F. Gurgan U. Aktan S. (2015). A study on social support and motivation. Anthropologist 22, 284–292. doi: 10.1080/09720073.2015.11891879

[ref57] Ushioda E. (2008). “Motivation and good language learners,” in Lessons from Good Language Learners. ed. Griffiths C. (Cambridge, UK: Cambridge University Press), 19–34.

[ref58] Vedder P. Boekaerts M. Seegers G. (2005). Perceived social support and well being in school; The role of students’ ethnicity. J. Youth Adolesc. 34, 269–278. doi: 10.1007/s10964-005-4313-4

[ref59] Wang Y. L. Derakhshan A. Zhang L. J. (2021). Researching and practicing positive psychology in second/foreign language learning and teaching: The past, current status and future directions. Front. Psychol. 12:731721. doi: 10.3389/fpsyg.2021.731721, PMID: 34489835 PMC8417049

[ref60] Wentzel K. R. (2009). “Peers and academic functioning at school,” in Handbook of Peer Interactions, Relationships, and Groups. eds. Rubin K. H. Bukowski W. M. Laursen B. (New York, NY: Guildford), 531–547.

[ref61] Wentzel K. R. (2016). “Teacher-student relationships,” in Handbook of Motivation at School. eds. Wentzel K. R. Miele D. B. (New York, NY: Routledge), 211–230.

[ref62] Wentzel K. R. Battle A. Russell S. L. Looney L. B. (2010). Social supports from teachers and peers as predictors of academic and social motivation. Contemp. Educ. Psychol. 35, 193–202. doi: 10.1016/j.cedpsych.2010.03.002

[ref63] Wigfield A. Eccles J. S. Fredricks J. A. Simpkins S. Roeser R. W. Schiefele U. (2015). “Development of achievement motivation and engagement,” in Handbook of Child Psychology and Developmental Science. eds. Lerner R. M. Lamb E. (New Jersey: John Wiley Sons), 657–700.

[ref64] Wigfield A. Wentzel K. R. (2007). Introduction to motivation at school: interventions that work. Educ. Psychol. 42, 191–196. doi: 10.1080/00461520701621038

[ref65] Wong L. H. Chai C. S. Chen W. Chin C. K. (2013). Measuring Singaporean students’ motivation and strategies of bilingual learning. Asia Pac. Educ. Res. 22, 263–272. doi: 10.1007/s40299-012-0032-2

[ref66] Yu S. Zhou N. Zheng Y. Zhang L. Cao H. Li X. (2019). Evaluating student motivation and engagement in the Chinese EFL writing context. Stud. Educ. Eval. 62, 129–141. doi: 10.1016/j.stueduc.2019.06.002

[ref67] Zhou X. (2020). Managing psychological distress in children and adolescents following the COVID-19 epidemic: A cooperative approach. Psychol. Trauma Theory Res. Pract. Policy 12, S76–S78. doi: 10.1037/tra0000754, PMID: 32551769

